# Interpersonal Coordination in Soccer: Interpreting Literature to Enhance the Representativeness of Task Design, From Dyads to Teams

**DOI:** 10.3389/fpsyg.2018.02550

**Published:** 2018-12-11

**Authors:** Rodrigo Santos, Ricardo Duarte, Keith Davids, Israel Teoldo

**Affiliations:** ^1^Núcleo de Pesquisa e Estudos em Futebol, Departamento de Educação Física, Universidade Federal de Viçosa, Viçosa, Brazil; ^2^Faculdade de Motricidade Humana, Universidade de Lisboa, Lisbon, Portugal; ^3^Football Association of Finland, Helsinki, Finland; ^4^Centre for Sports Engineering Research, Faculty of Health and Wellbeing, Sheffield Hallam University, Sheffield, United Kingdom

**Keywords:** soccer, interpersonal coordination, task representativeness, behavioral correspondence, action fidelity

## Abstract

Interpersonal coordination in soccer has become a trending topic in sports sciences, and several studies have examined how interpersonal coordination unfolds at different levels (i.e., dyads, sub-groups, teams). Investigations have largely focused on interactional behaviors at micro and macro levels through tasks from dyadic (i.e., 1 vs. 1) to team (i.e., 11 vs. 11) levels. However, as the degree of representativeness of a task depends on the magnitude of the relationship between simulated and intended environments, it is necessary to address a discussion on the correspondence between competitive and practice/experimental settings in soccer. The aims of this paper are to: (i) provide a brief description of the main concepts underlying the subject of interpersonal coordination in sports teams; (ii) demonstrate, through exemplar research findings, how interpersonal coordination in soccer unfolds at different scales; and (iii), discuss how coaches and researchers may ensure representativeness for practice and experimental tasks. We observed that papers addressing the analysis of interpersonal coordination tendencies in soccer often resort to dyadic (one vs. one) or sub-group (many vs. many) experimental tasks, instead of full-sized (11 vs. 11) games. Consequently, the extent to which such patterns reflect those observed in competition is somewhat uncertain. The design of practice and/or experimental tasks that rely on sub-phases of the game (e.g., 1 vs. 1, 4 vs. 4) should ensure the preservation of players’ behavior patterns in intended match conditions (11 vs. 11). This can be accomplished by measuring the level of action fidelity of the task, ensuring correspondence and successful transfer across contexts.

## Introduction

Coordination dynamics incorporates several concepts deemed relevant for investigating the intricate mechanisms that underpin performance behaviors in sports (Araújo et al., 2006). Analysis of sports performance through a dynamic perspective seeks to shed light on how the continuous interactions between elements of a system unfold at a microscopic level (i.e., player-player) and how they influence the emergence of macroscopic patterns (i.e., team collective structures) (Davids et al., 2005; Vilar et al., 2012). In order to address this issue, the investigation of interpersonal coordination tendencies in team games (e.g., soccer) has drawn the attention of sports scientists in recent years.

Research addressing the identification of the principles underpinning the interaction dynamics between players and teams has largely focused on behavior patterns at different system scales (i.e., dyads: 1 vs. 1, sub-groups: e.g., 3 vs. 3, teams: 11 vs. 11). Studies have uncovered the importance of collective variables – higher-level parameters that describe the emerging order of a system through the analysis of the functional behaviors of the elements that comprise this system (Kelso, 1995) – to interpersonal coordination at system and sub-system levels. However, it is still unclear whether the behaviors observed in dyadic tasks relate to those in competitive contexts, for instance (Duarte et al., 2012b; Clemente et al., 2013; Folgado et al., 2014). As the representativeness of a particular task refers to the correspondence between simulated and intended environments (Stoffregen et al., 2003), it is necessary to address a thoughtful discussion on this topic in soccer, as the utilization of tasks at the sub-system levels (e.g., small-sided and conditioned games) is widespread in training and research contexts (Araújo et al., 2007).

Therefore, the aims of this article are to: (i) provide a brief description of essential concepts about interpersonal coordination in sports teams; (ii) demonstrate, through exemplar research findings, how interpersonal coordination in soccer unfolds at different scales (i.e., dyads, sub-groups and teams); and (iii) discuss how coaches and researchers may ensure representativeness when designing practice and experimental tasks in soccer.

## Interpersonal Coordination

Over the years, several scientific fields have attempted to provide a clear definition of the term coordination, as it has been used to outline processes specific to particular disciplines. In animal physiology, for instance, coordination is regarded as “the processes involved in the reception of sensory information, the integration of that information, and the subsequent response of the organism” (Martin and Hine, 2008, p. 155). As for the subject of motor control, Bernstein (1967, p. 167) defined coordination as “the organization of the control of the motor apparatus,” i.e., how the multitude of micro-components of the system become coordinated into a controllable organization with the purpose of achieving task goals.

Hereupon, Schmidt et al. (1999) suggested that interpersonal coordination may be better understood through the mechanism of inherent self-organization, i.e., system tendencies that can be manipulated to generate order and structure within complex adaptive systems. Hence, the theoretical framework underpinning the function of such systems reveals that interpersonal coordination tendencies do not emerge uniquely from internalized cognitive or neural organization in individuals, but also from the way in which the elements of an organism interact. Furthermore, there is still evidence suggesting that interpersonal coordination can emerge subconsciously, even at an elite level of athletic performance (Stevens, 1976; Varlet and Richardson, 2015).

Kelso suggested that complex adaptive systems possess analogous structural features irrespective of their scales of function (Kelso, 1995). This is an important idea to guide performance in team sports, as it provides a theoretical foundation for understanding behaviors at all system levels. Accordingly, investigations have indicated that athletes’ behaviors need to be observed and understood in light of the same principles, both in individual and team sports competitions (McGarry and Franks, 1996; Grehaigne et al., 1997; Bourbousson et al., 2010). Supported by these principles, individual sports encompass inter-individual coupling between two opponents or between an athlete and an object/surface/element within the environment, whereas research on team sports individual system components, such as competing players, can entrain the behavior of the whole complex system, incorporating one or several dyads that combine to generate intra- and inter-team couplings (Bourbousson et al., 2010).

Correspondingly, the coupling interactions between oscillating elements in a collective system yields dynamic characteristics that equally describe the interactions in sports teams (McGarry et al., 2002; McGarry, 2005). Thus, systems and sub-systems in team sports (e.g., basketball, rugby union and soccer) are likely to display both competitive and cooperative characteristics. Consequently, every player within a team coordinates his/her actions with teammates in search for a solution for a joint performance purpose during competition. In addition, opposing players and teams must coordinate their actions between each other, in an attempt to create scoring opportunities for their respective sides and to prevent the opposition from scoring (McGarry et al., 2002; Glazier, 2010). Therefore, in team sports, analyses of performances of individual players and teams should take into account the influence of opponents on individual and collective actions (Duarte et al., 2012a). Hence, performance in team sports may be better understood as an outcome of the interpersonal relations between teammates and opponents and, consequently, such interactions should be considered inextricable for the analysis of match behaviors (Bourbousson et al., 2010).

With the purpose of identifying and describing the continuous, complex interactions between players and teams, a notable portion of the research on interpersonal coordination in soccer has largely analyzed the distinct interactional scales that comprise a complex adaptive system. This perspective considers all players as elements that oscillate around a mutual locus, and is based on the assumption that team vs. team interactions comprise numerous one vs. one interactions (McGarry, 2005).

## Interpersonal Coordination in Soccer – From Dyads to Teams

Soccer is a team sport with an essentially tactical nature, and thus demands from coaches, performance analysts and researchers an appropriate level of knowledge about the interactions between its elements (i.e., players and teams) (Davids et al., 2005). A soccer team can be described as a complex system whose interrelating elements generate a large variety of patterns at a macro scale, which differ from the micro-scaled behavior of each element considered in isolation (Passos et al., 2016b). Hence, the dynamic structures that comprise a soccer match should be contemplated in their entirety, rather than analyzed in a fragmented fashion because decomposing such deeply integrated systems for analysis can lead to artificial behaviors being observed (Grehaigne et al., 1997).

For this reason, seeking to understand how coordination between players and teams arises (and which are the resultant patterns), researchers have investigated interactions at various scales (between dyads – in 1 vs. 1 situations – sub-groups – attacking and defending units of players – and teams), in addition to proposing variables capable of explaining the emergence of collective behavior.

Thus, with the purpose of identifying these key collective patterns of behavior, some order parameters (e.g., surface area, centroids, distance to goal) were proposed, which addressed the analysis of these parameters from dyadic to team levels. In the following section, we attempt to provide a brief, though potentially thoughtful analysis of some of the studies that investigated one or more of these variables, according to the scale of analysis (i.e., dyads, sub-groups, and teams). In addition, we examine how the behavior of the collective variables proposed vary across the distinct scales.

### Dyads

Dyads (player–player interactions) are the basic unit of analysis for studying interpersonal coordination in team sports (Passos et al., 2015). Also, dyadic relations in competitive matches have been deemed essential to support the analysis of playing performance (McGarry, 2009). As a result, a considerable amount of research has sought to identify coordination patterns in attacker-defender dyads in team sports, particularly in soccer.

In team sports, athletes need to learn how to interact with teammates and opponents to achieve their goals (Gréhaigne and Godbout, 1995; McGarry et al., 2002). This process of continuous interaction is founded on players’ co-adaptive behaviors, which are constrained by locally created information (Passos et al., 2016a). This information emerges from different task constraints, including field markings and boundaries, and rules, which act as constraints on the co-positioning of teammates and opponents. Nevertheless, while markings and rules remain unchanged during competitive performance, players’ co-positioning in a match is continuously modified due to the presence and location of significant others. For example, studies in team sports (Passos et al., 2009; Correia et al., 2011) have revealed the relevance of key collective performance variables – such as territorial gain or the distance of the dyad to the scoring target – for the identification of dynamic patterns that emerge from dyadic relations.

Orth et al. (2014) investigated the characteristics of dyads in soccer, by examining the effects of defensive pressure on players’ running velocity during the approach to kick a football. Players were observed as they ran toward a football to cross it to a receiver in the penalty area who would try to score against a goalkeeper. Defensive pressure was manipulated in three conditions: without defenders; with a defender positioned far away; and with a defender positioned close to the player crossing the ball. Overall, findings suggested that the regulation of kicking is specific to a performance context and that some features of movement organization will not actually emerge unless the presence of a key information variable (e.g., a defender’s position) is manipulated as a task constraint during practice.

Headrick et al. (2012) examined how field location constrained the regulation of players’ movements. They sought to determine whether spatiotemporal relations between players and the ball in 1 vs. 1 sub-phases were constrained by their distance to the goal area. The experiment consisted of each participant performing the role of attacker (i.e., player in possession) and defender in settings designed to replicate actual match conditions. It was found that the modification of the dyads’ distance to goal influenced players’ behaviors and intentionality in relation to the ball. Specifically, they suggested that the variable “defender-to-ball distance” might be considered a critical collective variable, since the percentage of successful trials for the player in possession revealed higher success rates in positions closer to the goal.

### Sub-Groups

Although dyads represent the basic scale of interaction within the game, the analysis of more complex sub-phases has become relevant for understanding the coordination patterns emerging when players form groups (McGarry et al., 2002; Duarte et al., 2012a). Research (Passos et al., 2011; Headrick et al., 2012; Orth et al., 2014) suggest that, as small-sided and conditioned games can be viewed as a subscale of the formal game, these constrained game forms could be deemed useful for the investigation of structural and functional patterns in competitive settings.

Aiming to identify behavioral patterns in 3 vs. 3 sub-groups during creation and prevention of goal-scoring opportunities, Duarte et al. (2012b) analyzed group coordination through centroid (i.e., average team position) and surface area (i.e., occupied space) measures, obtained by manual video tracking procedures and 2-D reconstruction (Duarte et al., 2010). They reported that the centroids of both teams approached and moved away from each other’s defensive lines in a rather entwined (ebbing and flowing) manner, particularly at the moments that preceded the 3 vs. 3 system’s loss of stability (i.e., in attempts of goal assists). These findings suggest that both sub-groups moved synchronously in relation to each other. The emergence of such characteristics was influenced to a prominent degree by the distance between the attacking unit and the defensive line (Duarte et al., 2012b). On the other hand, the surface area did not reveal the existence of clear patterns of coordination between the teams. The fact that this measure did not uncover emergent patterns between sub-groups indicates that its utilization in small-sided and conditioned experimental and practice tasks, to understand and regulate collective behaviors in competitive contexts, demands further investigation. This is because sudden variations in this variable might occur due to more frequent turnovers of ball possession and increased player speed in the 3 vs. 3, in comparison to 11 vs. 11 contests (Duarte et al., 2012b).

Likewise, Frencken et al. (2011) aimed to identify emergent playing patterns in 4 vs. 4 SSCGs through centroid positions and surface area, acquired by positional data obtained through a transponder and antennas placed in a vest worn by players (Frencken et al., 2010). Results confirmed that teams’ centroid values display a tendency to move in the same directions. Also, findings revealed a stronger association between centroid forward-backward oscillations and lateral oscillations. Nonetheless, like Duarte et al. (2012b); Frencken et al. (2011) did not observe associations between teams’ surface area values. They attributed this outcome to the type of design of the experimental task. However, these studies raise doubts on whether the dynamics of some collective variables (e.g., surface area), analyzed in small-sided and conditioned games with a fewer players (e.g., 3 vs. 3, 4 vs. 4), correspond to those of a formal game.

### Teams

In soccer, some recent studies have been conducted in order to verify whether the patterns observed at lower scales (i.e., dyads and sub-groups) might also characterize behavior tendencies in competitive play.

For example, Frencken et al. (2012) examined whether the variability of inter-team distances related to critical periods of a match, as well as whether these periods were associated to key events (i.e., goals and goal attempts), by analyzing the variability of longitudinal and lateral distances between teams’ centroids. They acknowledged that, although in small-sided and conditioned games great variability of a positional measure often leads to key match events like goal-scoring opportunities, highly variable periods in inter-team distance values preceded only two out of fourteen goal attempts. Actually, results indicated that periods of high variability in inter-team distances emerged from changes in ball position. From this observation one may infer that ball dynamics in small-sided and conditioned games, particularly during crucial events, may differ from those observed in full-sized competitive matches. Also, it may be that inter-team distance (at least on the timescale analyzed by these investigators) may not be an accurate measure to capture the variability that precedes key match events in competitive contexts.

Also with respect to the variability of inter-team coordination, Vilar et al. (2013) analyzed emergent patterns of play in soccer, based on the assumption that locally outnumbering the opposing team is essential for defensive and offensive success. They utilized entropy measures (Shannon, 1948) to examine the uncertainty of the number of players of each team in sub-areas of play within what has been termed the effective-play space – an imaginary polygonal area with lines linking all outfield players located at the periphery of play at any given instant (Mérand, 1977). They observed that, in the course of a match, the teams seldom allocated more players than their opponents in sub-areas of play closer to the opposition goal. Moreover, entropy measures indicated that the central midfield sub-area of play was more unstable than all the other sub-areas, suggesting that numerical dominance within this sub-area is highly unpredictable. Nevertheless, as enlightening as these results may appear, such an approach has not been employed in the investigation of numerical instability at sub-system levels (i.e., dyads and sub-groups).

## Implications for Task Representativeness

Research on interpersonal coordination in soccer may potentially provide coaches with relevant information on how players and teams interact with each other, thus ensuring that skills acquired in practice are appropriately transferred to the competitive environment (Carling et al., 2005). Hence, the design of representative tasks – practice and/or experimental activities that preserve the unique properties of the intended environment (Hammond and Stewart, 2001) – is necessary to ensure successful transfer of individual and collective performances from learning/practice to competitive contexts (Pinder et al., 2011). However, the investigation of coordination patterns in soccer has often resorted to dyadic (one vs. one) and/or group (many vs. many) experimental tasks, and, consequently, the degree to which the interaction tendencies observed at these scales represent the behavioral dynamics in the intended environment is still somewhat uncertain (Bartlett et al., 2012).

This uncertainty is due to the fact that some studies, in an attempt to substantiate their findings, claim that the behavior of the variables that describe a given dynamic system comply with the same rules, regardless of the scale of analysis (i.e., dyads, sub-groups, and teams) (Frencken et al., 2011, 2012). However, these studies fail to acknowledge that the characteristics of each element or sub-unit can only be understood through the identification of the principles that describe the system at the ecological scale, rather than simply analyzing the properties of any given isolated parts (Capra, 1996). In addition, literature has already indicated that behavior in competitive contexts is highly dependent on situational constraints, particularly on the amount of system elements involved (Garcia et al., 2013; Passos et al., 2016a). Neglecting such constraints may result in loss of representativeness and, consequently, prompt emergent behaviors entirely different from those intended in competition.

In this respect, research data obtained in competition could certainly ensure action fidelity in practice/experimental tasks, through measurement and comparison of performance outcomes between both contexts. Action fidelity refers to the relation between the performance observed in a simulator (e.g., experimental or practice task), as well as in the intended system (e.g., team game) (Stoffregen et al., 2003). Therefore, designing practice tasks or test trials without taking into account the structural and functional correspondence with the competitive context may threaten the accurate interpretation of key aspects of performance, as well as the effectiveness of training activities and coaching interventions (Pinder et al., 2011). This is because in experimental and practice tasks, players may exhibit behaviors that do not resemble those displayed in a intended environment. This usually occurs when the sampling of constraints is not sufficiently thorough, and therefore does not enable the onset of certain perceptual demands (e.g., awareness of the possibility of a counterattack by the opposing team during a practice task) that are essential in competition (Araújo et al., 2007). Consequently, more research is necessary to validate appropriate measures of action fidelity (i.e., measures of task performance) that ensure that the essential behavioral characteristics inherent to the game will be preserved in practice and experimental contexts (Araújo et al., 2007).

Therefore, coaches and researchers should ensure that the dynamics of the intended environment (e.g., collective movement tendencies, relative strengths of both teams) are preserved, by comparing performance outcomes between practice/experimental settings and competition. Also, this approach could warrant that individual performances are reproducible (Stoffregen et al., 2003). Coaches could use this knowledge to design training activities that provide appropriate transfer of performance across training and competitive settings, thus ensuring that players are adequately adapted to constraints that are inherent to the sport itself, and not to a given random task.

In summary, the design of dyadic or sub-group experimental and practice tasks should account for both structural and functional characteristics of performance – which capture how players continuously regulate their behaviors in competitive settings. This approach should enable researchers and coaches to, respectively, increase the possibility for generalization of research findings and facilitate the application of acquired knowledge to performance in training contexts.

## Author Contributions

RS and RD drafted and wrote the paper. KD and IT wrote the paper.

## Conflict of Interest Statement

The authors declare that the research was conducted in the absence of any commercial or financial relationships that could be construed as a potential conflict of interest.
